# Residential Tetrachloroethylene Exposure: Response

**Published:** 2004-11

**Authors:** H. Kenneth Hudnell, Judith S. Schreiber

**Affiliations:** U.S. Environmental Protection Agency, Office of Research and Development, National Health and Effects, Research Laboratory, Neurotoxicology Division, E-mail: hudnell.ken@epa.gov; State of New York, Office of the Attorney General, Division of Public Advocacy, Environmental Protection Bureau, E-mail: judith.schreiber@oag.state.ny.us

We are grateful for the opportunity to respond to Storm and Mazor’s comments about our study “Apartment Residents’ and Day Care Workers’ Exposures to Tetrachloroethylene and Deficits in Visual Contrast Sensitivity” (Schreiber et al. 2002). We investigated potential relationships between environmental exposure to the dry-cleaning solvent perchloroethylene (perc, or tetrachloroethylene) and effects on visual function in two exposed populations (17 residents, including 4 children, in two apartment buildings and 9 adults working at a day care center) and age- and sex-matched control groups (*n* = 25 and *n* = 9, respectively). Mean airborne perc concentrations were 778 and 2,150 μg/m^3^ in the apartments and the day care center, respectively, levels well above the background range of < 1.6–22 μg/m^3^ [New York State Department of Health (NYSDOH) 1997]. Perc concentrations in biological samples were also elevated (Schreiber et al. 2002). We assessed visual function using tests of near acuity, near visual contrast sensitivity (VCS; a sensitive indicator of neurologic function), and color discrimination. Visual acuity did not differ between groups, but VCS scores from both the apartment residents and the day care workers were depressed across the spatial frequency spectrum, similar to results obtained in other solvent exposure studies (Broadwell et al. 1995; Campagna et al. 1995; Castillo et al. 2001; Donoghue et al. 1995; Frenette et al. 1991; Hudnell et al. 1996a; Mergler 1995; Mergler et al. 1991). We concluded that

Although the similar VCS deficits in both the residential study and day care investigation were apparently associated with chronic low-level environmental perc exposures, methodologic limitations preclude a definitive attribution of causation.

It is unlikely that age differences caused the group differences in VCS, as Storm and Mazor suggested. The exposed and control participants in the residential study were matched for age within 2 years, and the group means were within 1 year of each other. The mean age of the four exposed children was about 6 months greater than that of the six controls. The day care workers and controls were matched within 1 year of age, and the group means were within 6 months of each other. Such small age differences were highly unlikely to account for the VCS deficit.

Storm and Mazor reported that one exposed child was developmentally delayed and one had an attention deficit disorder, and they suggested that this may have caused the group difference in VCS. However, they did not provide comparable data for the control children, who were family members of NYSDOH employees. The same assessment of potentially confounding factors should have been applied to both groups. Furthermore, they cited a previously published article (Hudnell et al. 1996b) when suggesting that the VCS deficits in the exposed children may have been due to developmental delays. That article actually reported an association between perinatal exposure to airborne neurotoxicants and developmental delay in VCS (Hudnell et al. 1996b). We felt that it was inappropriate to exclude children from study participation because of conditions that may have been caused by perc exposure.

As noted by Storm and Mazor, “sample sizes were not sufficient to support statistical analysis of VCS stratified by age (i.e., child, adult)” in the residential study. It is not surprising that when they reduced the sample size to 13 pairs by excluding all children, the *p*-value increased from < 0.001 to 0.16, even though 7 of the 13 exposed adults had VCS scores in the lower 12th percentile of control scores.

We took several steps to minimize the influence of potentially confounding factors on VCS. A standard operating procedure and luminance control ensured test consistency. The exclusion criteria—failing to attentively complete the VCS test (one control resident excluded), having Snellen acuity worse than 20:70 (two eyes from exposed residents excluded, perhaps due to cataracts), and observing strabismus or other ocular anomalies (one control resident excluded)—were applied to both groups. None of the participants reported having an illness that might affect neurologic function. In the day care investigation, all participants were healthy females, and eight of nine were 21–29 years of age, thereby further reducing the potential for confounding. The observation of similar reductions in the VCS spatial-frequency profiles of the residential and day care exposed cohorts supported our conclusion that the effects may have been due to perc exposure.

Storm and colleagues recently conducted a study of apartment residents potentially exposed to perc and reported normal VCS in the exposed cohort (NYSDOH 1999, 2003). However, two factors limited comparability to our study (Schreiber et al. 2002). First, they measured far, rather than near, VCS. Near and far VCS do not provide comparable data due to differences in illumination, near and far visual acuity, and the visual field size of the test stimuli. Second, the mean airborne perc concentration was 34 μg/m^3^ in their study (NYSDOH 2003), 1–2 orders of magnitude lower than in our studies. These differences precluded an attempt to verify the VCS effects reported in our article (Schreiber et al. 2002). We stand by our methodologic procedures, results, and conclusions.
